# Rectus Sheath Block Using Bupivacaine Versus No Block for Postoperative Pain Management Following Midline Laparotomy: A Retrospective Comparative Study Using the Visual Analogue Scale

**DOI:** 10.7759/cureus.113735

**Published:** 2026-07-31

**Authors:** Garvit Singh, Jignesh P Dave

**Affiliations:** 1 General Surgery, Pandit Dindayal Upadhyay (PDU) Medical College, Rajkot, IND

**Keywords:** bupivacaine, midline laparotomy pain, postoperative pain management, rectus sheath block, regional anesthesia

## Abstract

Background

Effective postoperative pain management following midline laparotomy is critical to enhance recovery and minimize complications. The rectus sheath block (RSB) with bupivacaine has been proposed as a targeted, opioid-sparing regional analgesic technique. However, data from government hospital settings in India remain scarce. This study evaluated the efficacy of a single-dose RSB with 0.25% bupivacaine compared to standard postoperative analgesia using the Visual Analogue Scale (VAS).

Methods

A retrospective comparative study was conducted by reviewing records of patients who underwent midline laparotomy at Pandit Dindayal Upadhyay (PDU) Government Hospital, Rajkot, Gujarat, India, from January 2023 to June 2024 (1.5 years). Case records of 60 patients were reviewed and allocated to Group A (n = 30; RSB with 0.25% bupivacaine) or Group B (n = 30; standard care, no block) based on the analgesic technique documented in their operative and postoperative records. VAS pain scores, postoperative analgesia requirement, and rescue analgesia frequency, as recorded in nursing and anesthetic charts at 4, 8, 12, and 24 hours postoperatively, were extracted for analysis. Statistical analysis used the Mann-Whitney U test for VAS data and chi-square/Fisher exact tests for categorical outcomes.

Results

RSB significantly reduced VAS scores at all time points (median VAS: 3.00 vs. 5.00 at 4 hours; 4.00 vs. 6.50 at 8 hours; 4.00 vs. 7.00 at 12 hours; 5.00 vs. 7.00 at 24 hours; p < 0.001 at all time points). At four hours, none of the RSB patients required analgesia versus 25 (83.3%) in controls (χ² = 42.85, p < 0.001). Rescue analgesia was required four times by 25 (83.3%) controls versus none in the RSB group. Six (20.0%) RSB patients required no rescue analgesia over the 24-hour period.

Conclusion

Single-dose RSB with bupivacaine provides superior, prolonged postoperative analgesia in midline laparotomy patients, significantly reducing pain scores and analgesic requirements for up to 24 hours. RSB should be considered an integral component of multimodal postoperative pain management protocols, particularly where epidural analgesia is contraindicated or unavailable.

## Introduction

Midline laparotomy is one of the most frequently performed abdominal incisions in both emergency and elective surgery. Postoperative pain arising from this approach is typically severe and, if inadequately managed, predisposes patients to a cascade of complications including atelectasis, pneumonia, impaired respiratory mechanics, delayed ambulation, prolonged ileus, and extended hospital stay [1]. Achieving effective analgesia while limiting systemic, especially opioid-related, adverse effects remains a central challenge in peri-operative care.

Epidural analgesia has traditionally been considered the gold standard for postoperative pain control following major abdominal surgery [2]. However, its application in emergency laparotomy is frequently precluded by coagulopathy, hemodynamic instability, and sepsis [3,4]. Furthermore, the procedure demands specialist anesthetic expertise, and failure rates of 30%-50% have been reported [5]. These limitations necessitate safer and more accessible alternatives.

The rectus sheath block (RSB), first described in 1899 [6], involves depositing local anesthetic into the plane between the rectus abdominis muscle and its posterior sheath, blocking the anterior cutaneous branches of the thoracoabdominal intercostal nerves (T7-T12) [4]. This produces targeted somatic sensory blockade of the anterior abdominal wall without the hemodynamic risks associated with neuraxial techniques [4]. The technique has undergone significant evolution, from multiple blind injections in the 1980s to ultrasound-guided single-injection techniques since 2007 [7,8].

Bupivacaine, a long-acting amide local anesthetic discovered in 1957 and included on the WHO List of Essential Medicines [9], is the preferred agent for RSB owing to its prolonged pharmacokinetic profile. The relatively avascular nature of the rectus sheath compartment further slows systemic absorption, creating a depot effect that extends the duration of analgesia beyond that expected from the drug's intrinsic half-life [10,11].

Multiple randomized controlled trials, cohort studies, and meta-analyses have validated the analgesic efficacy of RSB in abdominal surgery [12,13]. However, comparative data in the context of emergency midline laparotomy at government hospitals in the Indian subcontinent remain limited.

This study was designed to compare VAS-measured postoperative pain scores and analgesic consumption between midline laparotomy patients receiving a single-dose RSB with 0.25% bupivacaine and those managed with standard analgesia alone, over the first 24 critical postoperative hours.

## Materials and methods

Study design and setting

A retrospective comparative study was conducted by reviewing case records of patients who underwent midline laparotomy at the Department of General Surgery, PDU Government Hospital, Rajkot, Gujarat, India, between January 2023 and June 2024 (1.5 years). Institutional Ethics Committee approval was obtained for the retrospective review of records, and the requirement for individual patient consent was waived as per institutional policy for retrospective record-based studies. All data were extracted exclusively from existing operative notes, anesthetic charts, nursing observation records, and discharge summaries.

Participants

Case records of 60 patients who underwent midline laparotomy (emergency or elective) during the study period were identified and reviewed. Patients were categorized into two groups based on the analgesic technique documented in their operative records: Group A (n = 30), patients who received RSB with 0.25% bupivacaine, and Group B (n = 30), patients who received standard postoperative care without a block. Group allocation was not randomized; the decision to administer RSB was based on the operating surgeon's preference at the time of surgery. All patients in both groups had received standardized preoperative analgesia, including intravenous fentanyl (1.5 mcg/kg body weight), under general anesthesia (GA), as documented in their anesthetic records. Group A additionally received RSB with 0.25% bupivacaine, while Group B received no additional regional block, in keeping with standard postoperative care.

The inclusion criteria applied during record review were as follows: all patients aged >12 years who underwent midline laparotomy for any indication, i.e., both emergency and elective procedures and both malignant and non-malignant pathology. Exclusion criteria were previous midline surgery; age <12 years; documented history of drug allergy; psychiatric illness; chronic strong opioid use; simultaneous stoma formation (colostomy/ileostomy); and known diabetes mellitus >10 years duration. Records with incomplete postoperative pain documentation at any of the four time points were also excluded. As no prospective screening log existed for this retrospective study, the exact number of records screened prior to exclusion could not be reliably reconstructed; all eligible records with complete postoperative pain documentation at all four time points (n = 60) were included in the final analysis.

RSB technique 

According to operative notes, the landmark technique was employed as routine clinical practice during the study period at our government hospital, where ultrasound guidance was not consistently available. During open laparotomy, needle position was additionally confirmed by direct intraoperative palpation, improving procedural safety. An 18-20G Tuohy needle was advanced perpendicular to the abdominal wall at the lateral edge of the rectus abdominis muscle in supine position. After skin penetration, the needle traversed the anterior rectus sheath (first tactile "pop") and was advanced until firm resistance of the posterior sheath was encountered [14]. In open procedures, needle tip position was confirmed by palpation from within the abdomen. Correct placement produces a distinctive "close but not too close" feeling on intra-abdominal palpation.

Operative records documented preparation of 50 mL of 0.25% bupivacaine by diluting 25 mL of 0.5% bupivacaine (5 mg/mL; 20 mL vials) with 25 mL of 0.9% normal saline, yielding a final concentration of 2.5 mg/mL. The maximum permissible dose was 3 mg/kg body weight. Injection was performed bilaterally, distributing equal volumes on each side [9].

Outcome measures and data extraction

Data were extracted from nursing observation charts and anesthetic follow-up records. The primary outcome was postoperative pain intensity as documented using the VAS (0 = no pain, 10 = worst possible pain) at 4, 8, 12, and 24 hours postoperatively [15]. Secondary outcomes extracted from records included (1) requirement for postoperative analgesia (yes/no) at each time point; (2) frequency of rescue analgesia administration over 24 hours; and (3) documented postoperative complications. Rescue analgesia had been administered when VAS > 4 or on patient request, as per the ward analgesic protocol in use during the study period.

Statistical analysis

Data were analyzed using IBM SPSS Statistics for Windows, Version 25.0 (Released 2017; IBM Corp., Armonk, NY, USA). Normality was tested using the Kolmogorov-Smirnov and Shapiro-Wilk tests. Given that VAS data were significantly non-normally distributed at all time points, the Mann-Whitney U test was employed for between-group VAS comparisons. Categorical data were compared using chi-square and Fisher exact tests as appropriate. A p value < 0.05 was considered statistically significant. Effect sizes were additionally calculated: rank-biserial correlation (r) for Mann-Whitney U comparisons and phi coefficient (φ) with odds ratio for chi-square comparisons, interpreted using conventional thresholds (0.1 small, 0.3 medium, 0.5 large)

As group allocation was not randomized, a sensitivity analysis was performed using propensity-score matching (1:1 nearest-neighbor, caliper 0.2 SD of the logit propensity score; covariates: age, sex) and inverse probability of treatment weighting (IPTW) with stabilized weights, to assess whether observed differences could be explained by baseline imbalance.

## Results

Demographic and baseline characteristics

Records of 60 patients (30 per group) were reviewed. The overall male-to-female ratio was 3.3:1, reflecting the predominantly male presentation to an emergency surgical unit. The mean age was 36.2 ± 17.8 years in Group A and 38.5 ± 15.4 years in Group B (t = 0.54, p = 0.59). Age distribution across seven decade-based categories is presented in Table 1. The Fisher's exact value for age distribution was 7.62 (p = 0.25), confirming no significant inter-group difference (Table 1). Sex distribution and ASA physical status are consolidated in Table 2. Chi-square testing confirmed that both groups were well-matched (χ² = 0.37, p = 0.76 for sex; χ² = 0.88, p = 0.64 for ASA status). These findings confirm baseline comparability between the two groups (Table 2). Premedication, antiemetic use, induction agent, intraoperative opioid, muscle relaxants, maintenance anesthesia, and NSAID administration were identical between groups per standard departmental protocol. RSB with 0.25% bupivacaine was the only intervention that differed between groups, confirming RSB as the sole differential exposure between Group A and Group B (Table 3).

**Table 1:** Age group-wise distribution of patients (N = 60) Fisher's exact value = 7.62, p = 0.25 (not significant). Both groups are comparable in age distribution. RSB: rectus sheath block.

Age group (years)	Group A (RSB), N (%)	Group B (control), N (%)
≤20	6 (60)	4 (40)
21-30	6 (46.2)	7 (53.8)
31-40	5 (50)	5 (50)
41-50	4 (40)	6 (60)
51-60	3 (30)	7 (70)
61-70	5 (100)	0 (0)
71-80	1 (50)	1 (50)

**Table 2:** Consolidated baseline demographic and clinical characteristics (N = 60) ASA: American Society of Anesthesiologists, RSB: rectus sheath block.

Variable		Group A (RSB)	Group B (control)	Test statistic	p-value
		N (%)	N (%)		
Sex	Male	24 (80)	22 (73.3)	χ² = 0.37	0.76
	Female	06 (20)	08 (26.7)		
Age (years)	Mean ± SD	36.2 ± 17.8	38.5 ± 15.4	t = 0.54	0.59
ASA physical status	ASA I	12 (40)	10 (33.3)	χ² = 0.88	0.64
	ASA II	13 (43.3)	14 (46.7)		
	ASA III	05 (16.7)	06 (20)		

**Table 3:** Intraoperative co-interventions and anesthetic protocol (N = 60) IV: intravenous, NSAID: nonsteroidal anti-inflammatory drugs, RSB: rectus sheath block.

Variable	RSB group (n = 30)	Control group (n = 30)
Premedication	Glycopyrrolate 0.2 mg IV	Same
Antiemetic	Ondansetron 4 mg IV	Same
Induction agent	Propofol 2 mg/kg IV	Same
Opioid (induction)	Fentanyl 2 µg/kg IV at induction	Same
Muscle relaxant (intubation)	Succinylcholine 1.5 mg/kg IV* or rocuronium 0.6-1 mg/kg IV^†^	Same
Maintenance anesthetic	Oxygen + air + sevoflurane/isoflurane	Same
Maintenance muscle relaxant	Vecuronium/atracurium as required	Same
NSAID	Diclofenac 75 mg IV (or paracetamol 1 g IV as per protocol)	Same
Additional opioid requirement	None routinely; rescue fentanyl only if clinically indicated	Same
Local anesthetic block	Bilateral rectus sheath block with 0.25% bupivacaine	Not administered

Postoperative analgesia requirement

Differences in analgesia requirement between the two groups were marked and statistically significant at all four time points. At four hours postoperatively, the earliest and clinically most critical assessment point, none of the RSB patients had required supplementary analgesia, compared to 25 (83.3%) patients in the control group (χ² = 42.85, p < 0.001). At eight hours, 8 (26.7%) from Group A versus 30 (100.0%) from Group B required analgesia (χ² = 34.73, p < 0.001). At 12 hours, 18 (60.0%) RSB patients versus 30 (100%) controls required analgesia (χ² = 15.00, p < 0.001). Even at 24 hours, the difference remained highly significant: 22 (73.3%) RSB patients versus 30 (100.0%) controls required analgesia (χ² = 9.23, p < 0.01) (Table 4).

**Table 4:** Comparison of postoperative analgesia requirement between both groups at each time point (N = 60) All differences were statistically significant (p < 0.001 at 4, 8, and 12 hours; p < 0.01 at 24 hours). RSB: rectus sheath block.

Time post-op	Group A (RSB); required analgesia, N (%)	Group A (RSB); no analgesia, N (%)	Group B (control); required analgesia, N (%)	Group B (control); no analgesia, N (%)	χ² value	p-value
4 hours	0 (0.0)	30 (100)	25 (83.3)	5 (16.7)	42.85	<0.01
8 hours	8 (26.7)	22 (73.3)	30 (100)	0 (0.0)	34.73	<0.01
12 hours	18 (60.0)	12 (40.0)	30 (100)	0 (0.0)	15.00	<0.01
24 hours	22 (73.3)	8 (26.7)	30 (100)	0 (0.0)	9.23	<0.01

Rescue analgesia frequency

The distribution of rescue analgesia frequency as extracted from nursing records (Table 5) further highlights the clinical superiority of RSB. In Group A, 6 (20.0%) patients required no rescue analgesia whatsoever over the 24-hour observation period, and a further 12 (40.0%) patients required it only twice, suggesting effective sustained analgesia beyond the initial block peak. In stark contrast, 25 (83.3%) Group B patients required rescue analgesia at all four time points (i.e., four times), and none required it fewer than three times. The Fisher's exact test yielded a highly significant value of 58.1 (p < 0.001), reflecting intergroup difference in pain burden. 

**Table 5:** Comparison of rescue analgesia frequency between both groups over 24 hours (N = 60) Fisher's exact value = 58.1, p < 0.001. The RSB group demonstrated substantially lower cumulative analgesic burden across the 24-hour observation window.

Rescue analgesia frequency (24 hours)	Group A (RSB), N (%)	Group B (control), N (%)	Clinical interpretation
None (0 times)	6 (20.0)	0 (0.0)	Complete analgesia; block fully effective
Once	7 (23.3)	0 (0.0)	Minimal breakthrough; block largely effective
Twice	12 (40.0)	0 (0.0)	Moderate; block adequate with gradual waning
Thrice	5 (16.7)	5 (16.7)	Block partially effective beyond 12 hours
Four times	0 (0.0)	25 (83.3)	Uncontrolled pain; no block effect

Postoperative complications were rare, occurring in 2 of 60 patients (3.3%). One RSB-group patient developed transient tachycardia and hypotension in the immediate postoperative period; one control-group patient developed a stitch-line infection. No cases of local anesthetic systemic toxicity, peritoneal puncture, vascular injury, or hematoma attributable to RSB were recorded in any patient's case file.

VAS score comparison

At four hours, the median VAS in Group A was 3.00 (IQR 1) versus 5.00 (IQR 2) in Group B (Z = -6.70, p < 0.001). At eight hours, median scores were 4.00 versus 6.50 (Z = -6.78, p < 0.001). At 12 hours, Group A maintained a median VAS of 4.00 versus 7.00 in Group B (Z = -6.76, p<0.001). At 24 hours, the intergroup difference remained statistically significant (median 5.00 vs. 7.00; Z = -6.77, p < 0.001). The gradual rise in Group A VAS scores from 3.00 at 4 hours to 5.00 at 24 hours reflects the expected pharmacological waning of a single bupivacaine dose, while Group B maintained consistently high scores throughout, peaking at a median of 7.00 (severe pain) from 12 hours onwards (Table 6).

**Table 6:** Comparison of VAS scores between both groups at all time points (N = 60)

Time post-op	Group A, median (IQR)	Group B, median (IQR)	Mann-Whitney Z	p-value
4 hours	3.00 (2-3)	5.00 (4-6)	-6.70	<0.001
8 hours	4.00 (3-4)	6.50 (6-7)	-6.78	<0.001
12 hours	4.00 (4-5)	7.00 (6-7)	-6.76	<0.001
24 hours	5.00 (4-5)	7.00 (7-8)	-6.77	<0.001

Effect sizes were calculated alongside significance testing. For VAS comparisons, rank-biserial correlation was large at all time points (r = 0.98 at 4 hours, 1.00 at 8 hours, 0.99 at 12 hours, and 0.99 at 24 hours). For analgesia requirement (Table 3), the phi coefficient likewise indicated large effect sizes at 4 hours (φ = 0.85), 8 hours (φ = 0.76), and 12 hours (φ = 0.50), and a medium-to-large effect at 24 hours (φ = 0.39). Corresponding odds ratios for requiring analgesia (RSB vs. control) were 0.004, 0.006, 0.024, and 0.043 at 4 hours, 8 hours, 12 hours, and 24 hours, respectively (Haldane-Anscombe correction applied at 4 hours, 8 hours, and 12 hours due to zero-count cells). These consistently large effect sizes indicate that the statistically significant differences reflect substantial clinical effect magnitudes rather than sample-size-driven significance.

Sensitivity analysis

Baseline covariates were well balanced before adjustment (standardized mean difference: age 0.04, sex 0.16). Propensity-score matching yielded 25 matched pairs with improved balance (SMD: age −0.18, sex 0.00); RSB patients had significantly lower VAS scores than matched controls at all time points (p < 0.001) and lower analgesia requirement at 4 hours, 8 hours, and 12 hours (p < 0.001), attenuating by 24 hours (p = 0.06). IPTW analysis of the full cohort corroborated these findings (VAS differences p < 0.001 at all time points; weighted risk differences for analgesia requirement significant at all time points, 95% CIs excluding zero). These results confirm primary findings are unlikely to be explained by age or sex imbalance between groups.

## Discussion

This prospective comparative study provides robust, statistically significant evidence that a single-dose RSB with 0.25% bupivacaine confers clinically meaningful advantages over standard postoperative analgesia in patients undergoing midline laparotomy. Benefits were evident from the first postoperative assessment at 4 hours and persisted across the 24-hour observation window. These findings are consistent with, and extend, several previously published studies [16-19].

The anatomical basis of RSB efficacy lies in the architecture of the anterior abdominal wall. The anteromedial abdominal wall is innervated by the terminal anterior cutaneous branches of the thoracoabdominal intercostal nerves (T7-T11) and the subcostal nerve (T12). These nerves travel in the neurovascular plane between the internal oblique and transversus abdominis muscles, pierce the posterior rectus sheath, and enter a potential space between the rectus abdominis muscle and the posterior sheath before finally piercing the muscle and anterior sheath to supply the skin. This intermediate potential space, rather than the posterior sheath itself, is the actual target plane for local anesthetic deposition in RSB [4,20]. By depositing local anesthetic within the potential space bounded by the posterior surface of the rectus muscle and the posterior sheath, RSB interrupts nociceptive transmission from the midline wound, ablating somatic afferent input without affecting visceral or sympathetic pathways. This selective somatic blockade accounts for both the analgesic efficacy and the absence of hemodynamic side effects associated with epidural techniques [21].

The pharmacokinetic basis for the prolonged analgesic effect, lasting well beyond the nominal 6-8-hour duration of bupivacaine, resides in the relatively avascular nature of the rectus sheath compartment. Slow systemic absorption creates a sustained local drug depot, maintaining effective tissue concentrations for extended periods [10,11]. This slower absorption kinetics is reflected in findings from Flack et al., who reported that time to peak plasma bupivacaine concentration following USGRSB was significantly longer than after wound infiltration (median 45 min IQR: 30-60 vs. 20 min IQR: 20-45, p = 0.006), attributing this delay to the reduced vascularity of the posterior rectus sheath compartment, restricted local anesthetic spread within this single muscle-layer contact zone [22].

The finding that none of the RSB patients required analgesia at four hours postoperatively, against 83.3% in the control group, reflects the importance of this early window. Elbahrawy and El-Deeb reported significantly lower VAS pain scores in the RSB group compared with control at two, four, and six hours postoperatively [23], along with reduced opioid consumption both intraoperatively and postoperatively, in patients undergoing laparotomy. Similarly, Bashandy and Elkholy reported significantly lower VAS scores with RSB compared to general anesthesia alone in patients undergoing midline abdominal cancer surgery, with reduced morphine consumption sustained not only in the immediate post-anesthesia period but throughout the first two postoperative days [24].

The reduction in rescue analgesia frequency carries substantial operational implications. In Group B, 83.3% of patients required around-the-clock supplementary analgesia at every four-hourly interval, imposing a considerable burden on nursing staff and increasing the risk of cumulative opioid toxicity. Opioid-related adverse effects, nausea, vomiting, respiratory depression, urinary retention, and postoperative ileus, are independently associated with prolonged hospital stay [25,26]. In Group A, 20% of patients required no rescue analgesia at all, and a further 63.3% needed it only once or twice, representing a clinically and economically significant reduction in analgesic burden. Cheung et al. highlighted the opioid-sparing potential of regional techniques as core to modern enhanced recovery after surgery (ERAS) protocols [25].

Compared with alternative regional approaches, RSB offers specific advantages for midline incisions. The transversus abdominis plane (TAP) block targets a similar nerve territory from a lateral approach but primarily provides lateral abdominal wall coverage, with less consistent midline analgesia [13,27]. A meta-analysis by Chin et al. found RSB potentially superior to TAP block for midline incision analgesia, including ease of catheter placement for continuous infusion [27]. Epidural analgesia, while effective, carries risks of epidural hematoma, abscess, and sympathetically mediated hemodynamic instability. Critically, RSB can be performed by the operating surgeon intraoperatively under direct vision, without specialist equipment or expertise, making it particularly relevant to district-level and government hospital settings.

The safety profile observed in this study is reassuring. No cases of local anesthetic systemic toxicity (LAST), peritoneal puncture, vascular injury, or hematoma were recorded. This accords with the systematic review by Rahiri et al., who found very low incidences of serious adverse events with RSB [13]. The open-abdomen approach used in this study, with palpation confirmation from within, provides an additional safety margin. Adherence to the weight-based maximum dose of 3 mg/kg bupivacaine is mandatory to avoid LAST.

The prolonged analgesic duration of RSB aligns well with ERAS recommendations, which emphasize effective and sustained peri-operative pain control as a prerequisite for early mobilization, earlier return of bowel function, and reduced hospital stay [28]. Future studies incorporating extended-release liposomal bupivacaine formulations, which have demonstrated analgesia lasting up to 72 hours in RSB [29], may further enhance outcomes in this setting.

Several limitations of this retrospective study merit acknowledgment. As data were extracted from existing hospital records, the possibility of documentation bias, incomplete records, or inconsistencies in pain score recording cannot be entirely excluded. The absence of a prospective patient screening log is a limitation of the retrospective design; the exact number of records excluded prior to identification of the final cohort could not be determined with certainty. The retrospective design precludes randomization and blinding, and unmeasured confounders may have influenced outcomes. Selection bias is an inherent limitation, as patients were not randomly allocated to treatment groups; group assignment reflected clinical decision-making at the time of surgery. The study was conducted at a single center with a predominantly male, emergency surgical population, which may limit generalizability. VAS was recorded at fixed four-hourly intervals as set by the ward analgesic protocol rather than being triggered by patient-reported pain episodes, potentially missing inter-assessment pain fluctuations between scheduled assessments. Additionally, nursing staff recording VAS scores were not blinded to group allocation, as the presence or absence of RSB was clinically apparent; this lack of blinding introduces a potential risk of performance and assessment bias in pain score documentation. Operator variability in performing the RSB technique was not assessed. Surgical duration was not consistently documented in the available records and could not be included as a comparator between groups. Formal measurement of time to first mobilization, length of hospital stay, opioid consumption in milligram equivalents, and patient-reported quality-of-recovery scores (e.g., QoR-40 [15]) were not systematically documented and could not be extracted. A future multicenter randomized, double-blind trial with balanced sex representation and comprehensive ERAS outcome metrics is warranted.

## Conclusions

Single-dose RSB with 0.25% bupivacaine appears to be a safe, technically accessible, and effective regional analgesic technique for patients undergoing midline laparotomy. Record review demonstrates statistically and clinically superior postoperative analgesia compared to standard care, with meaningfully lower pain scores and substantially reduced analgesic requirements sustained over 24 hours. Given the retrospective, non-randomized, single-center design of this study, these findings should be regarded as hypothesis-generating. The opioid-sparing effect and favorable safety profile suggest RSB as a promising candidate for multimodal postoperative pain management protocols, warranting confirmation through prospective, randomized trials before broader practice recommendations can be made.
